# New Drugs, Appliances, and Things Medical

**Published:** 1893-04-08

**Authors:** 


### NEW DRUGS, APPLIANCES, AND THINGS MEDICAL.

[All preparations, appliances, novelties, etc., of which a notice is desired, should be sent for the Editor, to care of The Manager, 428, Strand, London, W.C.]

### A NEW LAUNDRY SOAP.

Messrs. Stewart Brothers and Spencer's Oil Seed Mills, Rochester.

Good soaps for the laundry are of the highest importance. We are glad to be able to give Messrs. Stewart and Spencer's manufacture unqualified praise. Analysis shows that the soap contains nothing injurious to the most delicate materials but forms a compound of high excellence for cleansing purposes. Pure soda is non-existent, and the smallest amount only of the carbonate is present. It is especially valuable in the cleansing of woollen materials, and by its use the usual shrinkage will be avoided. Testimony from quarters where the practical tests have been applied enable us to pronounce on the popularity of the vegetable oil soap as an admirable cleansing medium and most agreeable in use.

